# Discovery and Partial Genomic Characterisation of a Novel Nidovirus Associated with Respiratory Disease in Wild Shingleback Lizards (*Tiliqua rugosa*)

**DOI:** 10.1371/journal.pone.0165209

**Published:** 2016-11-09

**Authors:** Mark A. O’Dea, Bethany Jackson, Carol Jackson, Pally Xavier, Kristin Warren

**Affiliations:** 1 School of Veterinary and Life Sciences, Murdoch University, Murdoch, WA, Australia; 2 Kanyana Wildlife Rehabilitation Centre, 120 Gilchrist Rd, Lesmurdie, WA, Australia; University of Texas Medical Branch at Galveston, UNITED STATES

## Abstract

A respiratory disease syndrome has been observed in large numbers of wild shingleback lizards (*Tiliqua rugosa*) admitted to wildlife care facilities in the Perth metropolitan region of Western Australia. Mortality rates are reportedly high without supportive treatment and care. Here we used next generation sequencing techniques to screen affected and unaffected individuals admitted to Kanyana Wildlife Rehabilitation Centre in Perth between April and December 2015, with the resultant discovery of a novel nidovirus significantly associated with cases of respiratory disease according to a case definition based on clinical signs. Interestingly this virus was also found in 12% of apparently healthy individuals, which may reflect testing during the incubation period or a carrier status, or it may be that this agent is not causative in the disease process. This is the first report of a nidovirus in lizards globally. In addition to detection of this virus, characterisation of a 23,832 nt segment of the viral genome revealed the presence of characteristic nidoviral genomic elements providing phylogenetic support for the inclusion of this virus in a novel genus alongside Ball Python nidovirus, within the *Torovirinae* sub-family of the *Coronaviridae*. This study highlights the importance of next generation sequencing technologies to detect and describe emerging infectious diseases in wildlife species, as well as the importance of rehabilitation centres to enhance early detection mechanisms through passive and targeted health surveillance. Further development of diagnostic tools from these findings will aid in detection and control of this agent across Australia, and potentially in wild lizard populations globally.

## Introduction

Wild reptile populations are vulnerable to a range of threatening processes including introduced and native predators[1], habitat modification [2], climate change [3], wildfires, anthropogenic trauma associated with urban environments (e.g. motor vehicle strikes) [4], and infectious diseases [5, 6]. Conservation impacts of infectious diseases in wild reptiles have been described in relation to translocation/reintroduction activities, through introduction of novel disease agents or disruption of disease dynamics within a population [5], as well as newly described or truly emerging pathogens [7, 8]. The continued advancement and availability of genomic analysis tools for detection of undescribed pathogens has markedly improved rapid detection of candidate aetiologic agents during observed outbreaks [9, 10], and should form a part of the toolkit for investigation of enigmatic and emerging wildlife disease syndromes.

A respiratory syndrome of shingleback lizards (“bobtails”, *Tiliqua rugosa*) in the Perth metropolitan region has been anecdotally described since the 1990s, largely based on admissions to Kanyana Wildlife Rehabilitation Centre (hereafter “Kanyana”) [11]. Observed clinical signs include excess mucous in the oral cavity, sneezing, serous to mucopurulent discharge from the eyes and nose, lethargy, inappetence, pale mucous membranes, depression, and loss of body condition. Colloquially termed “bobtail flu”, the term Upper Respiratory Tract Infection (URTI) was coined to describe this syndrome and thereby capture such cases on admission records. Clinical signs consistent with URTI are reportedly one of the main drivers for shingleback lizard admissions at Kanyana, alongside trauma due to domestic pets and motor vehicles [11]. As a result, a dedicated facility was built in 2004 to provide intensive care and quarantine capacity for affected individuals, and a treatment regime applied in the absence of a known aetiologic agent. This treatment consisted of a broad-spectrum antibiotic (Enrofloxacin, 10mg/kg, im), nebulisation with distilled water, an antiprotozoal (Metronidazole, 40mg/kg, oral), and supportive care including oral rehydration with 0.9% sodium chloride. Treatment success for affected individuals without comorbidities was 84% in 2014–2015 [11], however the fate of recovered and released individuals in the wild is unknown. The prevalence and epidemiology of respiratory disease in wild shingleback lizards, and thus the conservation implications, are also currently unknown.

Potential causes of respiratory disease in reptiles include infectious (bacterial, fungal, parasitic, viral) and non-infectious (trauma, neoplasia, environmental pollutants) factors [12]. Of the infectious causes of respiratory diseases in reptiles, viruses are well represented in the literature, partly due to the discovery of several new viruses of snakes and consequent review of the taxonomy [13]. Viruses associated with respiratory disease in captive reptiles include the ferlaviruses [13], Ball python nidovirus [14] and an Indian python nidovirus [9], atadenoviruses and reoviruses [15], ranaviruses [16], and Sunshine virus [10]. Viruses causing respiratory pathology and reported in both captive and wild reptiles include herpesviruses in chelonians [17], and reoviruses in snakes and lizards [18, 19]. Gram-negative bacterial respiratory infections are often described in captive reptiles, however are likely present as secondary or co-infections, and reflect the multi-factorial nature of disease in reptiles generally where environment, host and agent characteristics will result in a spectrum of clinical outcomes when individuals are exposed to aetiological agents [12]. There are few systematic and targeted epidemiological studies of respiratory diseases in wild reptile populations except in the case of mycoplasmosis (*Mycoplasma agassizii*) and herpesviral disease in chelonians, where there is a specific conservation management concern [20, 21]. There are few studies of respiratory disease in lizards, captive or wild, in the peer-reviewed literature, and most report small sample sizes or case series.

Shingleback lizards are large, robust lizards in the genus *Tiliqua*, commonly known as ‘bobtail’ lizards [22]. There are four sub-species found across Australia, three of which (*T*. *rugosa rugosus*, *T*. *rugosa asper* and *T*. *rugosa konowi*) are found in Western Australia [22]. This diurnal species has substantial variation in scale colour and pattern across its range, is a popular pet in Australia and overseas, and is also regularly seen in domestic environments where they appear to have adapted somewhat to urban encroachment. Whilst the species is considered common, they form a substantial caseload to urban rehabilitation centres from anthropogenic causes such as motor vehicle strike, dog and cat attack, and lawn mower trauma [11]. Several studies of lizards within the *Tiliqua* genus have documented ecto-, endo- and haemoparasites [23–26], and enteric bacteria [27], as well as the influence of their social networks and behavioural ecology on disease transmission [28]. However to date no viruses have been described in this species in Australia, captive or wild.

The order *Nidovirales* contains viral species with a single-stranded positive-sense RNA genome size range varying between approximately 13 and 33 kb, with a canonical gene structure noted in particular for the presence of two large open reading frames (ORFs) (1a and 1b) which occupy two-thirds to three-quarters of the 5’ end of the genome, and a ribosomal frameshift site (RFS) in the overlapping portion of ORF 1a and 1b [29]. The *Nidovirales* are classified into the families *Arteriviridae*, *Coronaviridae*, *Mesoniviridae* and *Roniviridae* [30]. The *Coronaviridae* family is represented by the *Coronavirinae* and *Torovirinae* subfamilies, encompassing a wide range of viral species infecting various mammalian, avian, piscine and, as recently identified, captive reptilian hosts [9, 14, 31]. These include significant zoonotic pathogens such as SARS and MERS coronaviruses [32, 33].

We describe here, for the first time, the systematic investigation of respiratory disease in shingleback lizards using next generation sequencing (NGS) technologies, and the discovery and partial characterisation of an associated nidovirus.

## Materials and Methods

### Sampling

All work in this study was carried out with Animal Ethics Committee approval from Murdoch University (permit number: R2753/15), and with the permission of the board of Kanyana. All samples were opportunistically obtained from shingleback lizards admitted to care at Kanyana, Western Australia, between April and December 2015. A case definition was established to assign individuals to a category of ‘case’, or ‘healthy’ on admission to Kanyana. Cases were individuals with serous or mucopurulent oculonasal discharge. Healthy individuals were those without clinical signs of upper respiratory disease, and in good to very good condition (excluding any traumatic injuries which resulted in their admission). Other clinical signs such as pale mucous membranes, sneezing, depression, or co-infections with faecal parasites were not included in the case definition, however were often noted on records. Cases were categorised initially by dedicated shingleback lizard rehabilitation staff, familiar with the signs of the respiratory syndrome and the case definition, and were then confirmed or re-classified by a wildlife veterinarian (author; BJ) according to the case notes from admission.

Samples were collected at the point of admission to the centre, prior to any treatment or mixing with other individuals. Strict asepsis was observed when collecting and handling individual animals and swabs, to avoid cross contamination. With the shingleback under manual restraint, a sterile neonatal flocked swab (FLOQSwabs^™^; Copan) was used to collect secretions from the oral cavity at the level of the glottis, and placed into Viral Transport Media (Medium 199 + Penstrep + Fungizone; Sigma). Samples were immediately stored at -20°C, and transferred to an -80°C freezer within two weeks. Individuals were treated according to their health status following sample collection (e.g. entered into the Kanyana treatment protocol for URTI, or treated for clinical signs on presentation).

### NGS and PCR screening

For initial nucleic acid extractions, samples of swab material from acute cases were pooled 1:5 before total nucleic acid from pools was extracted using a MagMax-96 Viral Isolation Kit (Ambion), according to the manufacturer’s instructions. As a control, a pool of healthy case material was processed in parallel. In order to capture potential RNA and DNA viral species for downstream NGS, total nucleic acid was converted to dsDNA via a combination of random priming and Klenow fragment based extension and PCR [34, 35]. Briefly, samples underwent reverse transcription using 1 μL primer NGS1random (CCTTGAAGGCGGACTGTGAGN_8_) at a concentration of 100 μM, 7 μL of purified sample, 10 μL of Protoscript II buffer (New England BioLabs) and 2 μL of Protoscript II First Strand cDNA enzyme (New England BioLabs) under the following reaction conditions: 25°C for 5 min, 42°C for 60 min and 95°C for 3 min. Complementary strand synthesis was performed by adding 2 μL of primer NGS1random (10 μM concentration) and 1 μL of Klenow polymerase (Promega) and incubating the reaction at 37°C for 1 hour. Double-stranded DNA products were then amplified using primer NGS1 (CCTTGAAGGCGGACTGTGAG) at a final concentration of 1 μM and AmpliTaq Gold 360 mastermix (Life Technologies) under the following conditions: denaturation at 95°C for 5 min, followed by 40 cycles of 95°C for 1 min, 55°C for 1 min, 72°C for 1min (increasing by 5 sec per cycle), and a final extension step of 72°C for 10 min. PCR product clean up was performed using a Wizard SV gel and PCR kit (Promega). Following PCR clean up, pooled products underwent library preparation and individual barcoding using a Nextera XT DNA library preparation kit (Illumina) according to the manufacturer’s instructions with the following minor modifications: during the NTA reaction, the cycling step of 55°C for 5 minutes was increased to 7 minutes and for the PCR bead clean-up, 30 μL of AMPure beads (Beckman Coulter) was used. Final libraries were pooled in equimolar amounts, and sequencing performed on an Illumina MiSeq using a V3 2x300 flowcell.

For conventional reverse transcription (RT) PCR, the primers BTnidoF (CGCGCAGAGTCATTTGACGTG) and BTnidoR (CCGACGATGATGATCTTTGCTGC) were developed based on an overlap sequence present in 5 of the contigs. These primers amplified a 391 bp product within ORF 1b. RT-PCR reactions were performed using One Taq One-Step reaction mix (New England BioLabs), 250 nM of each primer and 5 μL of RNA extract in a 25 μL total reaction volume. Reaction conditions were as follows, 48°C for 20 min, 94°C for 1 min, followed by 40 cycles of 94°C for 15 s, 55°C for 30 s and 68°C for 30 s, with a final extension step of 68°C for 7 min.

For quantitative RT-PCR, the primers LnF (CGGAGTGGACAAGTCGTGAA) and LnR (GGACTCAGTGCGGTGAGAAA), and the probe Lnprobe (FAM- CGTCGCCGGTCAGACAGCGAGCC-BHQ1) targeting a region of ORF 1a were developed. Reactions were performed using AgPath-ID One-Step reaction mix (Ambion), 400 nM of each primer, 120 nM of probe and 2 μL RNA extract (normalised to 10 ng/ μL) in a 20 μL reaction volume. Reaction conditions were as follows, 45°C for 10 min, 95°C for 10 min, followed by 40 cycles of 95°C for 15 s and 60°C for 45 s.

### Phylogenetic analysis

Read data was imported into CLC Genomics Workbench V7.5 (Qiagen) and demultiplexed, before *de novo* assembly was performed using CLC Genomics Workbench assembler with contig length set to a minimum of 1000 nucleotides. Contigs were then searched for homology to viral agents using BLASTn and BLASTx algorithms through the NCBI server (http://blast.ncbi.nlm.nih.gov/Blast.cgi), and using DIAMOND v0.7.1 [36]. Viral ORFs were predicted using Geneious v9.1.5, and the presence of conserved domains in the replicase polyprotein was analysed using InterProScan [37] and HHpred [38].

Phylogenetic analysis of the polyprotein 1ab full-length amino acid and RNA-dependent RNA polymerase (RdRp) domain sequences was performed using MEGA6 [39]. Sequences were aligned using MUSCLE, and maximum-likelihood (ML) trees estimated using the LG(+F) model [40] as selected by the best-fit substitution model ML analysis in MEGA. A discrete Gamma distribution was used to model evolutionary rate differences among sites. Reliability of the inferred trees was tested by the bootstrap method using 1000 replicates. Trees were rooted on the polyprotein 1ab gene or RdRp domain sequence of Cavally virus (GenBank accession no. YP_004598981). Sequence alignment of the entire polyprotein 1ab from uncategorised nidoviruses and members of the *Bafinivirus* and *Torovirus* genera was perfomed using MUSCLE, and similarity charts calculated based on the BLOSUM62 matrix [41].

## Results

### Sampling

A total of 83 individuals were entered into the study, including 48 ‘cases’ and 35 ‘healthy’ individuals based on assessment at admission. Reclassification by a wildlife veterinarian (author; BJ) according to detailed examination of the admission notes resulted in five cases being assigned as ‘healthy’, and six ‘healthy’ individuals being assigned as ‘cases’. Further, one individual considered healthy on admission was unable to be assigned as it was in poor body condition, however did not have clinical signs of oculonasal discharge. The resultant numbers for testing according to the case definition were therefore 49 ‘cases’ and 33 ‘healthy’ individuals (Table 1).

**Table 1:** Results of oropharyngeal swab testing for shingleback nidovirus 1.

Category	N	RT-PCR +ve	RT-PCR -ve	Prevalence (95% CI)	OR (95% CI)	p-value
**Case**	49	20	29	41% (27–56%)	5.0 (1.5–16.4)	0.006
**Healthy**	33	4	29	12% (3–28%)	NA	NA
**Uncategorised**	1	1	0	NA	NA	NA

Samples are from wild shingleback lizards admitted to Kanyana Wildlife Centre during 2015, according to case definition, with odds ratio (comparative factor being ‘healthy’ individuals) and p-value (Fischer’s exact 2-tailed test) for significance of associations.

### NGS and PCR screening

Following library preparation, six pools of case material (each containing up to 5 individual samples) and a single pool of healthy material (containing 5 samples) were available for testing. *De novo* assembly of MiSeq reads returned 17 individual contigs demonstrating varying levels of homology to polyprotein sequences of nidovirus species, but predominantly to the polyprotein 1ab (pp1ab) region of Ball python nidovirus (YP_009052475). Contigs with homology to nidoviruses were found in four of the six case pools. Two of the case pools and the healthy pool did not return any contigs with nidovirus homology. The smallest contig was 1109 nucleotides (nt) in length and the largest was 9534 nt in length. Via BLASTx search, multiple endogenous retrovirus sequences were found in all samples. A single pool had a small number of contigs return hits to Bearded dragon parvovirus NSP1 (Genbank accession YP_009154712.1), however mapping the reads back to this genome only resulted in 54 reads being mapped to a very short segment of approximately 80 nucleotides in the NSP1 region.

In order to obtain more of the putative nidovirus sequence by removing the dilution effect of pooling, three individual samples testing RT-PCR positive were prepared as above for MiSeq sequencing. This produced a further three contigs with sizes of 2639, 7739 and 13,813 nt demonstrating low-level homology to Ball-python nidovirus (KJ935003.1) with BLASTn query coverage and identity percentages of 7/79, 5/22 and 13/70 respectively. All putative nidovirus contigs from both MiSeq runs were then overlapped (minimum 50 bp overlap) to produce a single contig, followed by mapping all reads back to this contig, resulting in a final consensus viral sequence of 23,832 nt, with an average coverage of 3,255 (min 15x, max 19,535x). Raw read data from the four positive pooled samples and the three individual samples has been deposited in the NCBI Sequence Read Archive under accession numbers SAMN05761714—SAMN05761720.

All individual samples were tested via conventional RT-PCR using the primers BTnidoF/R, which were developed based on an overlap sequence present in 5 of the contigs. These primers amplified a 391 bp product, and the results of all testing are presented in Table 1. The odds of being RT-PCR positive for ‘cases’ was significantly higher than ‘healthy’ individuals (OR = 5.0, 95%CI: 1.5–16.4, *p =* 0.006) (Table 1). One individual that could not be assigned to our case definition was found to be RT-PCR positive. Normalised samples were tested by qRT-PCR to determine whether there was a difference in Ct values between ‘cases’ and ‘healthy’ individuals. The average Ct ‘cases’ was 33.98 and for ‘healthy’ 36.05 demonstrating less than log_10_ difference in viral concentration in tracheal swab samples across different groups. Testing of all RT-PCR negative samples using qRT-PCR confirmed their negative status.

### Genomic and phylogenetic analyses

**Fig 1:**
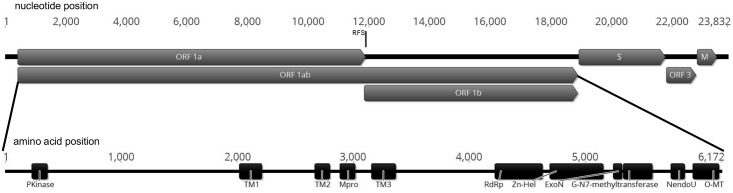
Partial genomic organization of Shingleback nidovirus 1. Nucleotide position is indicated along the top. Open reading frames (ORFs) and direction are represented by large block arrows. RFS indicates the position of the ribosomal frameshift site. Conserved nidovirus domains and their position within pp 1ab are indicated in an expanded view of the amino acid sequence below.

Analysis of the 23,832 nt viral contig revealed the presence of five large ORFs, with a genome organisation consistent with members of the *Nidovirales* (Fig 1) [29]. Using BLASTp homology results, these were designated as replicase ORF 1a, replicase ORF 1b, spike protein, putative accessory protein and membrane protein. The absence of a 3’ N protein encoding ORF and a terminal poly-A tail indicates this is likely to be a partial genome requiring further characterisation of the 3’ terminal sequence which may include further ORFs. Given the genomic organisation of nidoviruses, it may be that the 5’ coding region is complete, even if the 5’ termini are not completely mapped, however given the nearly 2000 amino acid difference between Ball python nidovirus pp1ab and shingleback nidovirus 1 pp1ab it must also be considered that there is an incomplete 5’ section of pp1ab. Also consistent with nidovirus genome organisation, is the presence of an overlap region between the large ORFs 1a and 1b of 94 nt, and within this region a putative ribosomal frameshift slippery sequence AAAAAAC [9].

A key feature of nidoviruses is the production of a large polyprotein, designated polyprotein 1ab (pp1ab). This is produced via a -1 ribosomal frameshift occurring at the slippery sequence resulting in a protein coded by both replicon ORF 1a and 1b [29]. Analysis of the pp1ab sequence from this viral contig resulted in a predicted 6172 amino acid protein.

Within pp1ab an ADP-ribose binding ‘macro’ domain, present in a number of members of the *Coronaviridae* was not detected, however various regions displaying similarities to protein domains of other nidoviruses were present in characteristic order. A protein kinase domain (PKinase) was detected, followed by a transmembrane domain (TM1) and then a further two transmembrane domains (TM2 and TM3) sandwiching the main protease (Mpro). These were followed by an RNA-dependent RNA polymerase (RdRp), a Zinc-helicase domain (Zn-Hel), a 3’-5’ exoribonuclease (ExoN), a Guanine-N7-methyltransferase (G-N7-methyltransferase), a uridylate specific endonuclease (NendoU) and a ribose-2’-*O*-methyltransferase (Fig 1).

The spike protein amino acid sequence was found to have 27% similarity to python nidovirus (AII00826) across 99% of the sequence, and the membrane amino acid sequence was found to have 37% similarity to Ball python nidovirus (YP_009052479.1) across 90% of the sequence. Analysis of the spike structure revealed a putative N-terminal signal peptide region and C-terminal transmembrane region separated by a long non-cytoplasmic domain. The size of the membrane protein is within the range reported for other nidoviruses, and the presence of three predicted transmembrane regions predominantly within the 5’ half is characteristic.

The 335 amino acid sequence encoded by ORF 3 (termed putative accessory protein) did not return any hits on BLASTp analysis. Attempts to predict function using ITASSER [42] and Phyre2 [43] were unsuccessful, with no significant templates found using either system. The protein is predicted to encode an N-terminal signalling region, a long non-cytoplasmic domain and a C-terminal transmembrane region, perhaps indicating that it is also a structural protein.

**Fig 2:**
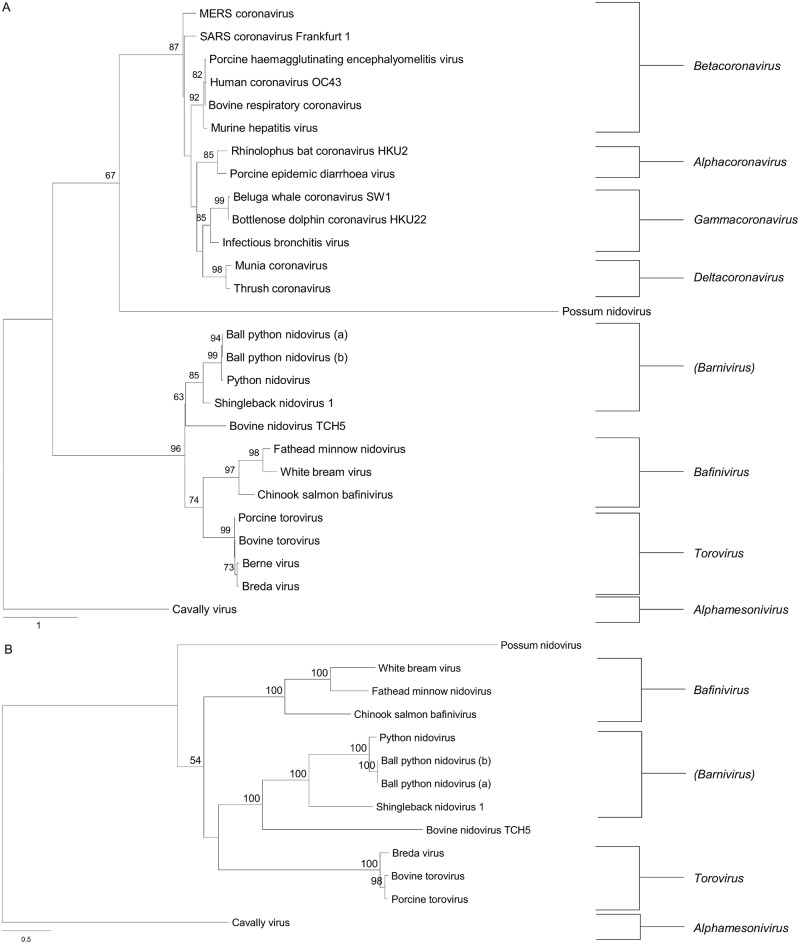
Maximum likelihood phylogenetic trees. (A) RdRp domain sequences of representative members of the *Coronaviridae*. (B) pp1ab sequences of members of the *Torovirus* genus, *Bafinivirus* genus, suggested *Barnivirus* genus and possum nidovirus. Trees are rooted on Cavally virus. The tree with the highest log likelihood is shown. Bootstrap support values are displayed above the branches. The tree is drawn to scale, with branch lengths measured in the number of substitutions per site. Viral GenBank accession numbers are as follows: Ball python nidovirus (a), AIM19602; Ball python nidovirus (b), YP_009052475; Beluga whale coronavirus SW1, YP_001876435; Berne virus, CAA36601; Bottlenose dolphin coronavirus HKU22, AHB63494; Bovine nidovirus TCH5, YP_009142787; Bovine respiratory coronavirus, ACT11016; Bovine torovirus, BAU21404; Breda virus, YP_337905; Cavally virus, YP_004598981.2; Chinook salmon bafinivirus, YP009130641; Fathead minnow nidovirus, ADN95978; Human coronavirus OC43, AAD32993; Infectious bronchitis virus, AAP92673; MERS coronavirus, AGN70927; Munia coronavirus, YP_002308505; Murine hepatitis virus, AAA46458; Porcine epidemic diarrhoea virus, AFC98503; Porcine haemagglutinating encephalyomelitis virus, AAD32992; Porcine torovirus, AIU41583; Possum nidovirus, AEU12347.2; Python nidovirus, AII00825; Rhinolophus bat coronavirus HKU2, ABB77027; SARS coronavirus Frankfurt 1, AAP33696; Thrush coronavirus, YP_002308496.1; White bream virus, YP_803213.

**Fig 3:**
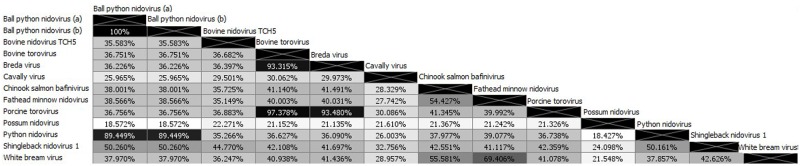
Pair-wise associations between viral pp1ab amino acid sequences in the *Torovirus*, *Bafinivirus* and proposed *Barnivirus* genera and possum nidovirus. Percentage level of similarity as calculated using the BLOSUM62 matrix is indicated. All GenBank accession numbers for viral sequences are listed in Fig 2.

Phylogenetic analysis of the full-length pp1ab amino acid sequence and conserved RNA-dependent RNA polymerase domain with representative members of the *Coronaviridae* family confirmed its position within the *Coronaviridae* family (Fig 2). In combination with the pairwise similarity analysis (Fig 3), the virus found in shinglebacks in this study appears most closely related to the recently described nidovirus species discovered in pythons, with the reptile-associated species forming a separate clade to toroviruses and bafiniviruses. Based on the current nomenclature, it is proposed that this virus be designated Shingleback nidovirus 1, and the sequence has been deposited in GenBank under the accession number KX184715.

## Discussion

This study describes a significant association between a novel virus found in oropharyngeal secretions, and cases of an enigmatic respiratory disease of wild shingleback lizards admitted to rehabilitation in the Perth region of Western Australia. The work highlights the utility of NGS in enabling rapid screening of pools of affected and unaffected individuals, to identify candidate pathogens for further investigation when baseline data is minimal, or previous diagnostics have failed to provide an answer. Findings from this study must be followed by diagnostic investigations including histopathology, immunohistochemistry, viral culture and experimental designs to provide evidence of causation [44], and to investigate whether this is a singular pathogen, or a component of a multifactorial disease syndrome as is commonly reported in reptile respiratory diseases [12, 20].

Genomic analysis of the virus discovered in this study demonstrates a number of features including large overlapping replicase genes, a ribosomal frameshift site, characteristic order of genes and size of the pp1ab, which is common in members of the *Nidovirales*. Analysis of the pp1ab conserved domains demonstrates features present in other nidovirus species including Ball python nidovirus [14], python nidovirus [9] and bovine nidovirus [45] were all present. In contrast to these viruses, a predicted Guanine-*N7*-methyltransferase domain could be identified based on Phyre2 analysis, spanning amino acid positions 5330–5587. This suggests that shingleback nidovirus 1 has potentially followed a different evolutionary pathway to the python nidoviruses, perhaps due to differences in host cells, where this capping enzyme has been lost [46]. It has also been shown, at least in mammalian systems, that this region has a role in virulence [47], and further mutagenesis studies will be required to determine if this is the case in reptiles. Phylogenetic analysis of this nidovirus shows that it clusters with the recently discovered Ball python nidovirus and python nidovirus, which are also associated with severe respiratory disease [9, 14, 31]. The similarity across the pp1ab between the putative Shingleback nidovirus 1 and the python nidoviruses of approximately 50% is greater than that between Shingleback nidovirus 1 and members of the *Bafinivirus* and *Torovirus* genuses (approximately 42% and 43% respectively) and supports the proposal by Stenglein et al. (2014) that a new genus, *Barnivirus*, be formed. This proposal was based only upon the existence of Ball python nidovirus, however the results of this study would indicate that Shingleback nidovirus 1 and python nidovirus would also be members of the proposed *Barnivirus* genus.

Although we found a significant association between cases of respiratory disease and the RT-PCR detection of the novel nidovirus, we also found RT-PCR positive individuals that were apparently healthy, and case-positive individuals that were not RT-PCR positive. There are likely several reasons for this. Our case definition may lack sensitivity and/or specificity with potential misclassification due to the reliance on visual assessment of clinical signs. We minimised observer bias by using the same rehabilitation staff member (author: CJ) to assign cases, and following up with a review of the case history by a wildlife veterinarian (author: BJ) to corroborate classification. If the respiratory disease is caused by the nidovirus discovered, it is unknown what the incubation period or shedding rates and routes are, and therefore the sensitivity/specificity of the RT-PCR detection method used for screening individual samples in this study is also unknown. Thus there is potential for false negatives where individuals were either pre-clinical, not shedding, or samples were below detection limits for the RT-PCR. It is recommended those surveying shingleback lizards in captivity or wild for this respiratory disease capture key clinical signs including mucous membrane colour, presence and type of oculonasal discharge (mucopurulent/serous), body condition, weight and snout-vent length, and include other diagnostics where possible such as haematology, biochemistry, and radiographic and histopathological studies. It is also possible that this is a multifactorial syndrome, and while the presence of the novel nidovirus may increase the chances of shingleback lizards developing respiratory disease, there may also be a requirement for, as yet, uncharacterised co-factors or co-infectious agents. Further work should include investigation of bacterial species in affected and unaffected individuals, noting the importance of collecting samples from affected tissues to ensure any species detected are representative of pathologic processes rather than normal respiratory flora [48].

There is evidence the described respiratory syndrome is widespread in wild shingleback lizards of the Perth region, as well as anecdotal reports of a similar syndrome in these lizards in other states of Australia. A study of *Tiliqua rugosa* from South Australia reported ocular and nasal discharge in wild individuals, with haematological evidence of a chronic infectious process [49]. Given the severity of recently described nidoviral disease syndromes in other species such as pythons [31] and brushtail possums [50], coupled with the high caseload and mortalities without treatment reported by wildlife care centres in the Perth metropolitan region, it is critical to determine the distribution and impact of this virus in wild shingleback lizards and related genera. Further work needed includes confirming a causal link between Shingleback nidovirus 1 and the respiratory syndrome, identifying and describing any co-infections including bacterial pathogens, an epidemiological analysis of admissions to wildlife centres to identify risk factors for admission, and ongoing development of diagnostic tools for screening of captive and wild shingleback lizards. This targeted surveillance should include a spatiotemporal design and *Tiliqua* species from areas with distinct geographic boundaries or isolated populations, as this will help infer the relatedness or endemicity of the virus, seasonal influences on viral prevalence, and any host-pathogen evolutionary relationships that may influence disease ecology [51, 52]. More widespread screening of related genera and sympatric lizard species is also important to determine if there are alternate species that may act as reservoirs or spillover hosts.

Historically, viral causes of disease in reptiles were likely to be under-reported for many reasons including the cost and bioinformatics support required to investigate novel diseases using NGS technologies, as well as co-infections with microbial species, and poor sampling techniques that obscure the true aetiologic agent [6]. Pathogen discovery tools, which are not reliant on targeting a specific organism, are increasingly being used in wildlife disease investigations [10, 17, 53, 54], although uptake is considered slow comparative to health research in humans and domestic animals [54]. Thus we anticipate an increase in the number of viruses detected in wildlife taxa that have had few viral disease investigations, such as lizards, compared to well-studied mammals such as bats, rodents and primates where their capacity to host zoonotic pathogens arguably provides public health drivers for research as well as funding incentives [55, 56]. A relative lack of baseline information on normal viruses as well as host ecology in wild reptiles, can limit epidemiological interpretation of infectious disease findings for these species. However as baseline data expands, concurrent with the increasing availability and decreasing costs of molecular tools, there should be a greater depth of understanding of the significance of detected pathogens for the conservation management of captive and wild species.

The detection of this novel nidovirus in association with a long-reported respiratory syndrome demonstrates the importance of wildlife centres, as well as zoos and private clinics, in providing passive disease surveillance data and samples for early detection of emerging wildlife diseases and monitoring of existing diseases [57–59]. Ongoing collaborative arrangements between researchers and wildlife rehabilitation centres are critical to detect and describe these novel infectious disease agents. The development of a qRT-PCR to screen for the novel virus as part of this study will now enable rapid screening of shingleback lizards and related lizard groups to determine host species and geographic spread, in captivity and the wild.
